# Development of Monoclonal Antibodies for Detection of Conserved and Variable Epitopes of Large Protein of Rabies Virus

**DOI:** 10.3390/v13020220

**Published:** 2021-01-31

**Authors:** Wen Zhao, Jingyin Su, Naiyu Zhao, Jie Liu, Shuo Su

**Affiliations:** Jiangsu Engineering Laboratory of Animal Immunology, Institute of Immunology and College of Veterinary Medicine, Nanjing Agricultural University, Nanjing 210095, China; 2017207031@njau.edu.cn (W.Z.); xiao_anny@yeah.net (J.S.); 17117420@njau.edu.cn (N.Z.); 2016107063@njau.edu.cn (J.L.)

**Keywords:** rabies virus, large protein, monoclonal antibody, epitope

## Abstract

Rabies virus (RABV) causes fatal neurological encephalitis and results in approximately 6000 human death cases worldwide every year. The large (L) protein of RABV, possessing conserved domains, is considered as the target for detection. In this study, three monoclonal antibodies (mAbs), designated as 3F3, 3A6 and L-C, against L protein were generated by using the recombinant truncated L protein (aa 1431–1754) and the epitopes were also identified using a series of overlapping truncated polypeptides for testing the reactivity of mAbs with different RABV strains. The ^1479^EIFSIP^1484^, ^1659^RALSK^1663^ and ^1724^VFNSL^1728^ were identified as the minimal linear epitopes recognized by mAbs 3F3, 3A6 and L-C, respectively. Amino acid alignment showed epitope ^1724^VFNSL^1728^ recognized by mAb L-C is completely conserved among RABV strains, indicating that mAb L-C could be used to detect all of the RABV strains. Epitope ^1479^EIFSIP^1484^ is highly conserved among RABV strains except for a P1484S substitution in a China I sub-lineage strain of Asian lineage, which eliminated the reactivity of the epitope with mAb 3F3. However, the epitope ^1659^RALSK^1663^ was only completely conserved in the Africa-2 and Indian lineages, and a single A1660T substitution, mainly appeared in strains of the China I belonging to Asian lineage and a Cosmopolitan lineage strain, still retained the reactivity of the epitope with mAb 3A6. While both A1660T and K1663R substitutions in a China I lineage strain, single K1663R/Q substitution in some China II strains of Asian lineage and some Arctic-like lineage strains and R1659Q mutation in a strain of Africa-3 lineage eliminated the reactivity of the epitope with mAb 3A6, suggesting mAb 3A6 could be used for differentiation of variable epitopes of some strains in different lineages. Thus, variability and conservation of the three epitopes of L protein showed the reactive difference of mAbs among RABV strains of different lineages. These results may facilitate future studies in development of detection methods for RABV infection, the structure and function of RABV L protein.

## 1. Introduction

Rabies is an ancient zoonosis posing threat to human health for centuries. Over 150 countries, especially the countries in Africa and Asia, are still troubled by rabies (https://www.who.int/). Rabies virus (RABV) is usually inoculated from the saliva of infected animals to healthy animals including humans, and then retrogradely transported to the central nervous system (CNS), resulting in acute, fatal encephalomyelitis [1]. Rabies is still an incurable disease which urges the need to further elucidate the detailed antigenicity/pathogenicity of viral proteins and mechanism of virus replication. RABV belongs to the genus *Lyssavirus*, family *Rhabdoviridae*, order *Mononegavirales*. The non-segmented negative-strand genome of RABV is composed of five genes encoding the nucleoprotein (N), phosphoprotein (P), matrix (M) protein, glycoprotein (G), and large (L) protein in the following order: 3′-N-P-M-G-L-5′. The phylogenetic pattern based on the L gene is similar to the phylogenetic pattern based on the N gene [2], which was highly conserved in RABV. The L protein was reported to be the target for detection of RABV [3].

The RABV L protein has multiple functions in viral replication and pathogenicity. Both the polymerase L and P protein binding with DLC1 (dynein light chain 1) influences microtubule organization of and mediates cytoskeleton reorganization, this helps to the viral components transport within cells for primary RABV transcription [4]. The L protein together with P protein shaping the P-L polymerase complex and constitutes the viral ribonucleoprotein (RNP) complex with NP protein, which is essential for L protein to function as a viral RNA-dependent RNA polymerase (RdRp), and then manipulate RABV replication and transcription [5,6]. The first 19 amino acid residues of the P protein and a motif close to the carboxy terminus of the L protein are critical to form the P–L complex [7]. The fragment ^1929^NPYNE^1933^ located in the positions aa 1562 to 2127 in carboxy terminus of L protein was reported to be critical region for the interaction between L protein and P protein, which thus influenced the RdRp activity [8]. The L protein has GDP polyribonucleotidyltransferase (PRNTase) activity that could promote viral mRNA cap formation, and the amino acid residues 1112 G, 1170T, 1201 W, 1241 H, 1242 R, 1285 F, and 1286 Q are critical for viral mRNA capping [9]. The RABV strains with K1685A and K1829A mutation of L protein were highly attenuated, indicating that the L protein is of significant importance in viral pathogenicity [10]. Together with RNP, G protein could induce stronger virus-neutralizing antibody (VNA) production and trigger equal protective immunity response as the whole virus, which is possibly explained by that RNPs may activate the cellular immune response for the enhanced VNA production [11].

In this study, we aim to generate RABV-specific monoclonal antibodies using the purified truncated L protein (aa 1429–1757) of RABV CVS-11 strain as the immunogen, and subsequently define the linear epitopes recognized by the generated mAbs.

## 2. Materials and Methods

### 2.1. Cells and Virus Strains

BHK-21 cells and SK-N-SH cells (human neuroblastoma cells) were sourced from ATCC (American Type Culture Collection). BHK-21 cells were cultured in the Dulbecco’s modified Eagle’s medium (DMEM) (HyClone, Logan, Utah, USA) which contains 10% fetal bovine serum (Gibco, California, USA) and 1% Penicillin-Streptomycin (NCM Biotech, Suzhou, Jiangsu, China). SK-N-SH cells were cultured in the Minimum Essential Medium (MEM) (HyClone, Logan, Utah, USA) which contains 10% fetal bovine serum (Gibco, California, USA) and 1% Penicillin-Streptomycin (NCM Biotech, Suzhou, Jiangsu, China). The challenge virus standard RABV CVS-11 strain (GenBank no. GQ918139) and the vaccine strain HEP-flury (GenBank no. AB085828) were kept in our laboratory at −80 ℃ and propagated in SK-N-SH cells. The virus titer was tested using 50% tissue culture infectious dose (TCID_50_) by the Reed-Muench method [12] and the RABV-infected cells were detected by immunofluorescence assay (IFA).

### 2.2. Generation of mAbs

The truncated L protein (aa 1429–1757) of RABV in prokaryotic expression system could induce the antibody response efficiently by immunizing mice, suggesting that this fragment had good antigenicity [13]. Thus, we select the fragment as antigen to prepare mAbs against L protein. The aa 1431–1754 fragment with His-tag was expressed in a prokaryotic expression system and mainly accumulated in insoluble fraction. Then the recombinant and truncated His-L protein (rtL-his) were further purified by nickel-nitrilotriacetic acid (Ni-NTA) Agarose. Six-week old female Bagg albino (BALB/c) mice (6 mice each group) were first immunized subcutaneously with 100 μg of purified rtL-his protein emulsified with an equal volume of Freund’s complete adjuvant (Sigma-Aldrich, St. Louis, MO, USA) and thereafter with an equal mix of rtL mixed Freund’s incomplete adjuvant (Sigma-Aldrich, St. Louis, MO, USA) every 2 weeks for two times. The mice with the best immune response were further boosted with the purified rtL-His before fusion. The hybridomas were generated by fusion of the immunized splenocytes with myeloma cells SP2/0 using the polyethylene glycol 2000 (Sigma-Aldrich, St. Louis, MO, USA). Hybridomas were seeded and cultured in the Roswell Park Memorial Institute (RPMI) 1640 medium (HyClone, Logan, UT, USA) containing hypoxanthine-aminopterin-thymidine (HAT) (Sigma-Aldrich, St. Louis, MO, USA) in 96-well plates for two weeks. The clones were screened using indirect enzyme-linked immunosorbent assays (Indirect ELISA). Positive clones were then subjected to subcloning three more times through limiting dilution to establish stable antibody-secreting cells, which were intraperitoneally injected into paraffin-sensitized mice to generate mice ascites. The reactivity of mAbs with virus protein was test by western blot and Indirect immunofluorescence assay (IFA).

### 2.3. Indirect Enzyme-Linked Immunosorbent Assays

Hybridoma screening was carried out by indirect Enzyme-Linked Immunosorbent Assays (ELISA). Briefly, the purified rtL protein was diluted with carbonate buffer (pH 9.6) to 2 μg/mL, coated in ELISA plates (Nunc, Glostrup, Denmark) and placed at 4 °C overnight. The plates were then washed three times with phosphate buffer saline (PBS) containing 0.05% Tween-20 (PBST). After washing with PBST three times, the culture supernatants of hybridomas and SP2/0 cells (as negative control) were incubated in 96-well plates at 37 °C for 1 h. Thereafter, the plates were washed again and incubated with 1:10,000 diluted horseradish peroxidase (HRP) -conjugated goat anti-mouse IgG (KPL, Gaithersburg, MD, USA) for 1 h at 37 °C. After washing, the 3,3′,5,5′-tetramethylbenzidine (TMB) was added and reacted at 37 °C for 10 min. Then, 50 µL of H_2_SO_4_ (2 M) were used to terminate the reaction. The antigen-antibody binding analysis was conducted using automatic ELISA plate reader (TECAN, Männedorf, Switzerland) by measuring the optical density value at 450 nm.

### 2.4. Isotypes Identification of mAbs

Subtypes of generated mAbs were classified using the mouse monoclonal antibody isotyping kit (Biodragon Immunotechnologies, Beijing, China). Briefly, ELISA plates were first coated with purified rtL protein at 4 °C overnight. After washing once with PBST, the culture supernatants of hybridomas were added and the plates were incubated at 37 °C for 30 min. The plates were washed five times with PBST and then incubated with the HRP-conjugated goat anti-mouse Ig (G1/G2a/G2b/G3/M/A/κ/λ) for 30 min at 37 °C. After washing five times, the TMB was added and then incubated at 37 °C for 10 min. The well was showing blue color (positive) and the HRP-conjugated antibody used in the blue well indicates the subtype of mAbs.

### 2.5. Western Blot

BHK-21 cell samples infected with CVS-11 or HEP-flury, rtL-His protein or truncated fragments of CVS-11 L protein were separated by sodium dodecyl sulfate polyacrylamide gel electrophoresis (SDS-PAGE) and then transferred from polyacrylamide gel onto nitrocellulose (NC) membranes (GE Healthcare, Amersham, UK). After blocking with 5% non-fat milk. The NC membranes were washed with PBST three times and then incubated with the diluted ascites fluid (1:1000) at 37 °C for 1 h. After washing three times, the 1:10,000 diluted HRP-conjugated goat anti-mouse antibody (KPL, Gaithersburg, MD, USA) was used as the secondary antibody and incubated at 37 °C for 1 h. Then the membranes were washed again and exposed to the enhanced chemiluminescence (ECL) buffer (Vazyme, Nanjing, China), subsequently were detected by Amersham imager 600 (GE Healthcare, Amersham, UK).

### 2.6. Immunofluorescence Assay

BHK-21 cells were infected with CVS-11 or HEP-flury, then incubated for 72 h. The adherent cells were fixed with cold methanol-acetone (1:1) for 2 h at −20 °C following washing with PBS. Subsequently, the cells were incubated with the hybridomas culture supernatants, SP2/0 culture supernatants (negative control), or anti-N mAb (positive control) produced in our laboratory [14]. After washing, the 1:500 diluted Fluorescein isocyanate (FITC)-conjugated goat anti-mouse IgG (KPL, Gaithersburg, MD, USA) was used as the secondary antibody. Immunofluorescent was captured using a fluorescent microscope (Nikon, Tokyo, Japan).

### 2.7. Epitope Mapping

**Figure 1:**
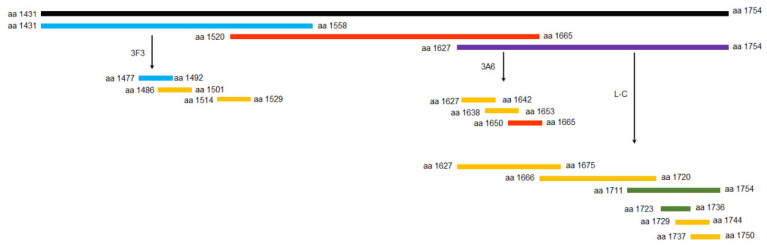
Schematic representation of the truncated L fragments used for epitopes screening. The black fragment represents the region used to express the recombinant truncated L (rtL), blue fragment represents the regions reacting with mAb 3F3, red fragment represents the regions reacting with mAb 3A6, green fragment represents the regions reacting with mAb L-C, purple fragment represents the regions reacting with mAb 3A6 and mAb L-C, and yellow represents fragments not reacting with any mAbs.

The rtL gene was first truncated into three overlapping fragments (Figure 1) and cloned into the pET-32a (+) vector with 6× His tag. Primers used for amplification are listed in Table 1. When the polypeptide that reacted with the antibody was approximately 10 amino acids in length, amino acids at the C-terminus and N-terminus were reduced one by one alternately or simultaneously until the smallest polypeptides recognized by the mAbs were identified. The complementary primer pairs and the primers used for amplying fragments with mutant epitope are listed in Table 2. They were synthesized, annealed, and further cloned into the pGEX-4T-1 vector (*Eco*RI/*Xho*I) with GST tag. The His- or GST-fused polypeptides were both induced expression in *E. coli* BL21 (DE3). All the reactivity of fusion polypeptides with mAbs were analyzed using Western blot.

### 2.8. Spatial Conformation and Variation Analysis of Epitopes

To understand the spatial position of the identified epitopes in the three-dimensional structure of the RABV L protein, we used the polymerase complex (PDB ID: 6UEB) as a template to model and visualize it using the PyMOL software. The hydrophilicity, antigenic index and the surface probability were analyzed using the PROTEAN software (DNASTAR, Madison, WI, USA). The coding regions of the L gene of different RABV strains were retrieved from Genbank and a phylogenetic tree was constructed using RAxML (v8.2.10) under the General Time Reversible (GTR) + GAMMA model. The amino acid of L protein was also compared among different RABV strains using the Molecular Evolutionary Genetics Analysis (MEGA) 7.0.26 software.

## 3. Results

### 3.1. Generation of mAbs against RABV L Protein

**Figure 2:**
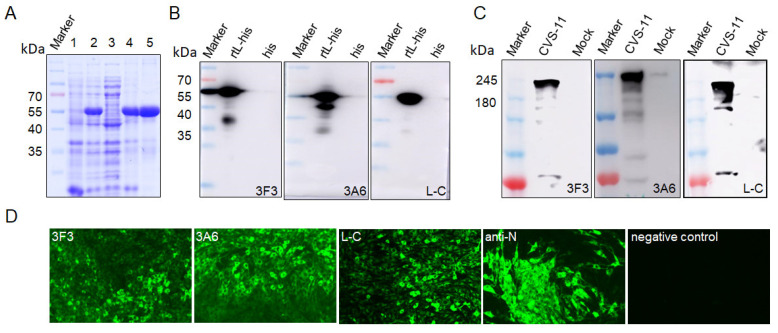
The reactivity of the generated mAbs with recombinant truncated L protein (rtL-his) and CVS-11 strains. (**A**) SDS-PAGE analysis of expression of rtL-his. Lysates from *E. coli* BL21 (DE3) transformed with plasmid pET-32a without isopropylthio-β-galactoside (IPTG) induction (lane 1) and pET-32a-rtL (lane 2) with IPTG induction, cell supernatant (lane 3) and the insoluble fraction (lane 4) of *E. coli* BL21 transformed with IPTG-induced pET-32a-rtL after sonication; lane 5, purified his-rtL. (**B**) MAbs 3F3, 3A6 and L-C were used to detect rtL-his expressed in in *E. coli* BL21 by western blot. MAbs 3F3, 3A6 and L-C were used to detect L protein in BHK-21 cells infected with CVS-11 by western blot (**C**) and indirect immunofluorescence assay (IFA) (**D**).

The expression of the truncated L protein (aa 1431–1754), designated as rtL, of CVS-11 strain (GenBank no. GQ918139) were detected by SDS-PAGE, and the result showed an expected protein band of 57 kDa, suggesting that the rtL-His protein was successfully expressed with a His-tag (Figure 2A). The rtL-His was purified for further immunization in mice. The antibody titers of the mice immunized with truncated L protein detected by indirect ELISA were higher than 10^−5^, indicating the recombinant truncated protein effectively induced an immune response. Three hybridomas stably secreting mAbs against the L protein (designated as 3F3, 3A6, and L-C) were generated through fusing, screening, and sub-cloning. Western blot showed that the mAbs 3F3, 3A6, and L-C could bind rtL-His with a protein band of 57 kDa (Figure 2B). The CVS-11, which were cultured in BHK-21 cells, was used to test the reactivity of the three mAbs using western blot. The result showed that mAbs 3F3, 3A6, and L-C could bind an approximately 240 kDa of protein, respectively, which was about the same size with the L protein of RABV (Figure 2C). In addition, the three mAbs showed strong reactivity with BHK-21 cells infected with the CVS-11 by immunofluorescence assay (IFA), whereas no florescent signal was detected using the supernatant of sp2/0 myelomas as the primary antibody (Figure 2D). These results revealed that the mAbs showed good reactivity to RABV virus. Subtype analysis demonstrated that the heavy chain of mAbs 3F3, 3A6 and L-C was IgG1, and the light chain was the κ subtype.

### 3.2. Mapping of the Linear Epitopes of the L Protein

**Figure 3:**
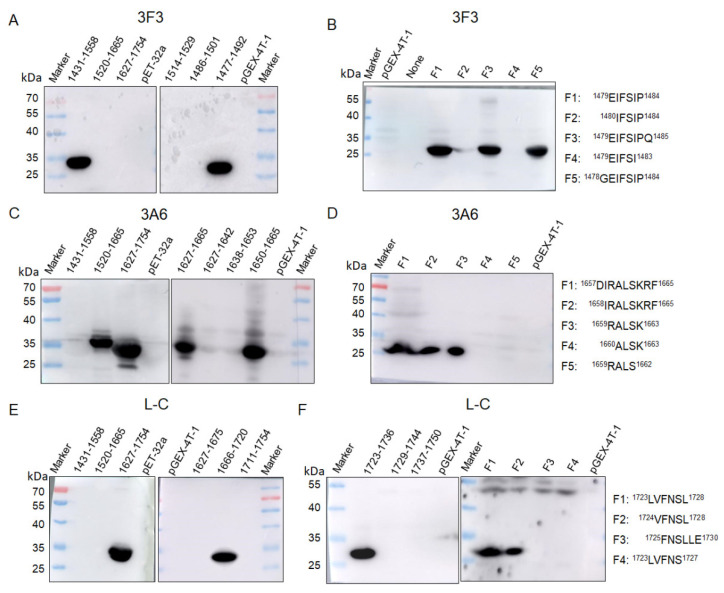
Mapping of epitopes recognized by mAbs 3F3, 3A6 and L-C by western blot. (**A**,**B**) Truncated fragments were detected with mAb 3F3 by western blot and proteins containing ^1479^EIFSIP^1484^ were recognized by mAb 3F3. (**C**,**D**) Truncated fragments were detected with mAb 3A6 by western blot and proteins containing ^1659^RALSK^1663^ were recognized by mAb 3A6. (**E**,**F**) Truncated fragments were detected with mAb L-C by western blot and proteins containing ^1724^VFNSL^1728^ were recognized by mAb L-C.

To identify the linear epitopes of L protein, the rtL protein was truncated to three overlapping segments (aa 1431–1558, aa 1520–1665, and aa 1627–1754) (Figure 1) fused to the His-tag and expressed in *E. coli* BL21 (DE3). Western blot showed that mAb 3F3 and mAb L-C could react with the fragment (aa 1431–1558) and the fragment (aa 1627–1754), respectively (Figure 3A,E), while mAb 3A6 showed reactivity with both aa 1520–1665 and aa 1627–1754 (Figure 3C), implying that the epitope recognized by mAb 3A6 was located in the region of aa 1627–1665. Furthermore, the overlapping segments aa 1627–1642, aa 1638–1653, and aa 1650–1665 fused with the GST-tag were used for the second round of screening, and mAb 3A6 could only react with aa 1650–1665 by western blot (Figure 3C). Thus, aa 1650–1665 were divided into five polypeptides and three polypeptides (^1657^DIRALSKRF^1665^, ^1658^IRALSKRF^1665^ and ^1659^RALSK^1663^) could be bound by mAb 3A6, while mAb 3A6 showed no reactivity with the other two polypeptides ^1659^RALS^1662^ and ^1660^ALSK^1663^ suggesting that ^1659^RALSK^1663^ was the minimum linear epitope of L protein recognized by mAb 3A6 (Figure 3D). To quickly identify the epitopes recognized by mAb 3F3 and L-C, the linear epitopes on aa 1431–1558 and aa 1627–1754 was predicted using the ABCpred server (http://crdd.osdd.net/raghava/abcpred/index.html). Three predicted epitopes (aa 1514–1529, aa 1486–1501, and aa 1477–1492) containing 16 amino acids in aa 1431–1558 were tested for the reactivity with mAb 3F3 by western blot and mAb 3F3 only showed reactivity with aa 1477–1492 (Figure 3A). Furthermore, five polypeptides of aa 1477–1492 were tested for the reactivity with mAb 3F3. The result showed that three polypeptides (^1479^EIFSIP^1484^, ^1479^EIFSIPQ^1485^, and ^1478^GEIFSIP^1484^) could be recognized by mAb 3F3, while mAb 3F3 showed no reactivity with polypeptides ^1480^IFSIP^1484^ and ^1479^EIFSI^1483^ (Figure 3B). Thus, ^1479^EIFSIP^1484^ was the minimum linear epitope of L protein recognized by mAb 3F3. Using the similar strategy, the minimum linear epitope of L protein recognized by mAb L-C was identified as ^1724^VFNSL^1728^ (Figure 3E,F).

### 3.3. Spatial Location of the Defined Epitopes

**Figure 4:**
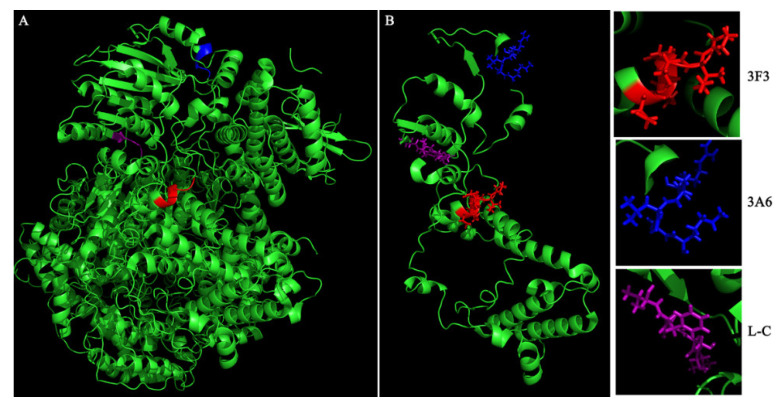
Spatial localization of the epitopes recognized by mAbs 3F3, 3A6 and L-C in L protein. The spatial structure of RABV polymerase complex (PDB ID: 6UEB) was visualized by PyMOL software. Epitopes recognized by mAbs 3F3, 3A6 and L-C are marked with red, blue, and purple respectively. Spatial localization of the identified three epitopes in the complete polymerase complex (**A**) and the aa 1431–1754 fragment of L protein (**B**).

**Figure 5:**
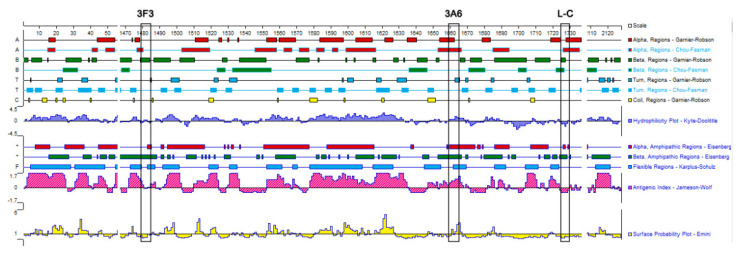
Antigenic, hydrophilicity and the surface probability index of the defined epitopes. The index was predicted using the PROTEAN software and three epitopes are shown in boxes.

We used the structure of the polymerase complex of RABV SAD B19 strain (PDB ID: 6UEB) as a template to model the defined epitope domains of L protein. As shown in Figure 4, the epitopes ^1479^EIFSIP^1484^ recognized by mAb 3F3 (marked with red) and ^1659^RALSK^1663^ recognized by mAb 3A6 (marked with blue) were both fully exposed on the surface of L protein, while epitope ^1724^VFNSL^1728^ recognized by mAb L-C (marked with purple) was partially exposed on the surface and partially buried by the surrounding protein structure. Besides, the hydrophilicity, antigenic index and the surface probability of the identified three epitopes were analyzed in the PROTEIN software. When comparing with epitopes ^1479^EIFSIP^1484^ (recognized by mAb 3F3) and epitope ^1724^VFNSL^1728^ (recognized by mAb L-C), ^1659^RALSK^1663^ (recognized by mAb 3A6) possessed high hydrophilicity, high antigenic index and highsurface probability (Figure 5).

### 3.4. Conservation and Variation Analysis of Identified Linear Epitopes.

**Figure 6:**
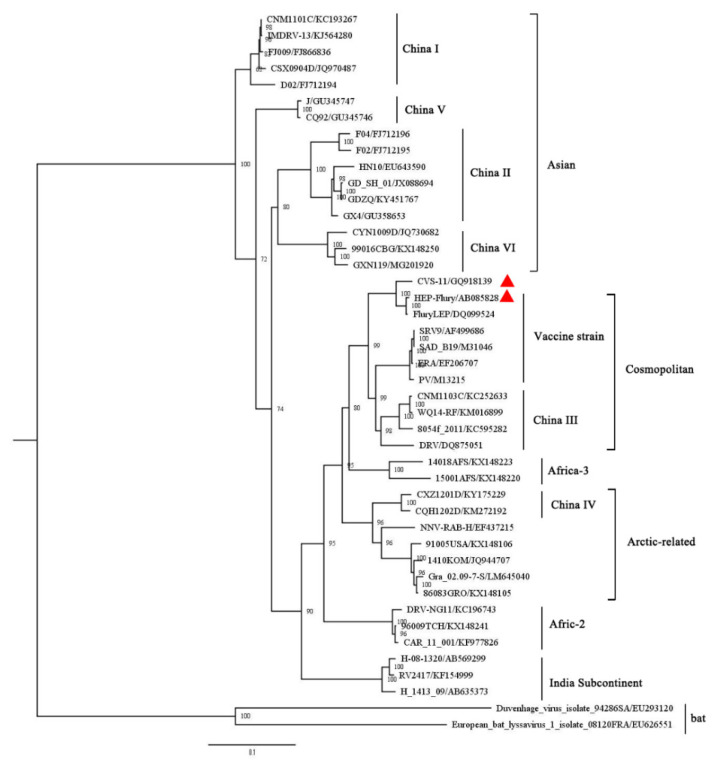
Phylogenetic tree constructed based on the RABV L gene. The RAxML (v8.2.10) was used to constructed the maximum likelihood (ML) tree based on the GTR + GAMMA model. The bootstrap value was calculated based on 1000 replicates. The strains used in this study are marked with red triangles. The GenBank number of individual RABV strain are listed behind the “/” symbol.

A maximum likelihood phylogenetic tree was constructed based on 43 complete nucleotide sequences of L gene. Different RABV strains were divided into six lineages of Asian, Cosmopolitan, Arctic-related, Indian subcontinent, Africa-2, and Africa-3 [15] (Figure 6). In addition, the China (I, II, V and VI) sub-lineages are classified into the Asian lineage, China III belongs to Cosmopolitan, and China IV belongs to the Arctic-related lineage [16] (Figure 6). The CVS-11 used in this study clusters in the Cosmopolitan lineage (Figure 6).

**Figure 7:**
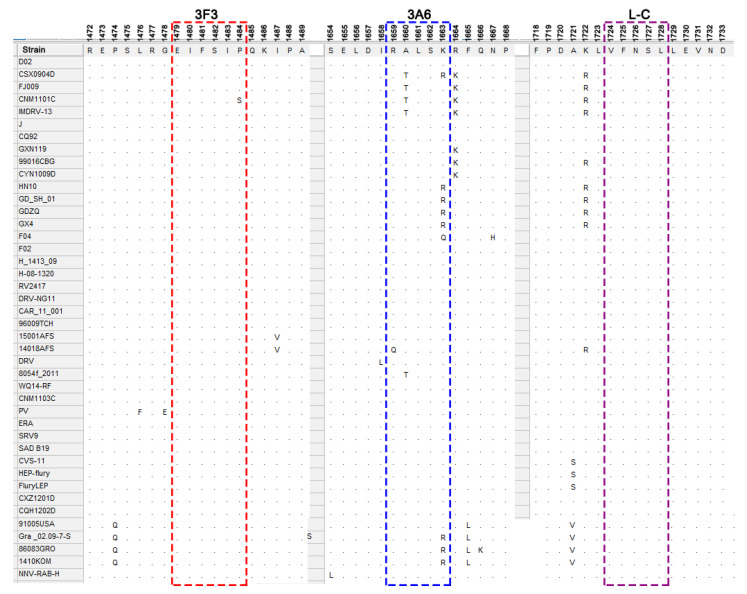
Conservation analysis of the defined epitopes. Amino acid alignment was conducted by the MEGA (7.0.26) software, and the identified minimal epitopes ^1479^EIFSIP^1484^, ^1659^RALSK^1663^ and ^1724^VFNSL^1728^ are shown in boxes. Amino acids in the location of epitope that are consistent with the epitopes are indicated by dots, while the amino acids that are not consistent with the epitope sequence are shown in abbreviations of amino acids (such as S, Q, T and R).

To evaluate the variation of the defined epitopes among different strains, amino acid sequences of L protein among different lineages of RABV strains were aligned using the MEGA 7.0.26 software. The amino acid alignment showed that epitope ^1724^VFNSL^1728^ recognized by mAb L-C is completely conserved among RABV strains, indicating that mAb L-C could be used to detect all of the RABV strains. Except for a P1484S substitution in CNM1101C strain of the sub-lineage China I of Asian lineage, the epitope ^1479^EIFSIP^1484^ recognized by mAb 3F3 is highly conserved among different lineages. In comparison, the epitope ^1659^RALSK^1663^, recognized by mAb 3A6, was only completely conserved in the Africa-2 and Indian lineages, and four substitutions (R1659Q, A1660T, K1663R, or K1663Q) within the epitope appeared in some strains of the other lineages (Figure 7). R1659Q mutation happened in 14018AFS strain belonging to Africa-3 lineage. Single A1660T substitution was mainly distributed in strains of the China I sub-lineage belonging to Asian lineage and a strain of the China III sub-lineage belonging to Cosmopolitan lineage. K1663Q mutation fell in a F04 strain of China II sub-lineage, while single K1663R substitution appeared in some strains in the Arctic-related lineage and China II sub-lineage of Asian lineage. Both A1660T and K1663R substitutions fell in a CSX0904D strain in the China I sub-lineage of Asian lineage in phylogenic tree. Notably, vaccine strains were distributed into Cosmopolitan lineage and had no amino acid substitutions on these three epitopes (Figure 7).

**Figure 8:**
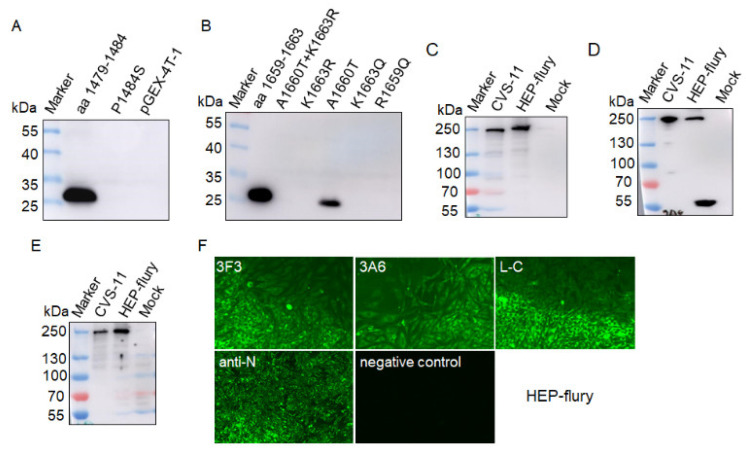
The reactivity of mAbs against mutant epitope fragments and HEP-flury. (**A**) The mutation P1484S eliminated the reactivity of epitope ^1479^EIFSIP^1484^ with mAb 3F3. (**B**) Single substitution A1660T was recognized by mAb 3A6; R1659Q, K1663R, and K1663Q eliminated the reactivity of mAb 3A6 with the epitope ^1659^RALSK^1663^. MAbs 3F3, 3A6 and L-C were used to detect L protein in BHK-21 cells infected HEP-flury by western blot (**C**–**E**) and IFA assay (**F**).

To further explore whether these substitutions of amino acid residues influenced the reactivity of mAb, mutant epitopes (aa 1479–1484 and aa 1659–1663) of L protein of CVS-11 were used to test the reactivity of mAb 3F3 and mAb 3A6, respectively. Western blotting assay showed the P1484S mutation eliminated the reactivity of the epitope ^1479^EIFSIP^1484^ with mAb 3F3 (Figure 8A), suggesting mAb 3F3 could be used for differentiation of strains with P1484S mutation. For the epitope ^1659^RALSK^1663^, only the epitope with the A1660T substitution could be recognized by mAb 3A6, while a single mutation R1659Q, K1663R or K1663Q eliminated the reactivity of mAb 3A6 with the epitope ^1659^RALSK^1663^ (Figure 8B), suggesting mAb 3A6 could be used for differentiation of variable epitopes of some strains in different lineages. Further, we used the vaccine strain HEP-flury in the Cosmopolitan lineage to test the reactivity with these three mAbs, the results showed these three mAbs showed good reactivity with HEP-flury in western blot and IFA assays (Figure 8C–F), which was consistent with conservation of the three epitopes in the vaccine strains (Figure 7).

## 4. Discussion

Rabies has threatened human health for centuries. The RABV L protein is a multi-functional protein responsible for virus transcription, replication [4,6,9], and virus pathogenicity [10]. RABV L protein was also reported as the potential target for diagnosis [3]. Here, we immunized mice with purified truncated L protein (aa 1429–1757) of CVS-11, which was reported to efficiently induce the antibody response in mice [13], and generated three mAbs against L protein, designated as 3F3, 3A6 and L-C. All of the mAbs could recognize the RABV L protein of CVS-11 by IFA and western blot, showing good reactivity for RABV (Figure 2D). RABV is an ancient virus and causes an incurable disease, thus the detection and monitoring of rabies virus (RABV) is still very important. Many researchers have reported many mAbs against RABV N [17,18], P [19], G [20] and M [12] proteins. And some epitopes of P [21] and M [12] proteins of RABV were identified and could be used to evaluate the reactivity of mAbs to different virus strains by analyzing their conservation and variability. Previous study has produced the polyclonal antibodies against the L protein by using the recombinant truncated L protein (aa 1429–1757) [13], which showed good reactivity with RABV strains and could be used to detect the virus. Compared with polyclonal antibodies, monoclonal antibodies have better specificity for detection [22]. In this study, three mAbs (3F3, 3A6, and L-C) against L protein were first generated and further the exact location of epitopes recognized by the three mAbs were mapped by expressed truncated L protein in *E. coli* and analyzed by western blot. Epitope mapping indicated that polypeptide ^1479^EIFSIP^1484^ recognized by mAb 3F3, ^1659^RALSK^1663^ recognized by mAb 3A6 and ^1724^VFNSL^1728^ recognized by mAb L-C were identified as the minimal linear epitopes in the L protein.

The mAbs generated in this study could react with CVS-11 strain and HEP-flury (vaccine strain). Unfortunately, we did not have enough strains to test the reactivity of the three mAbs and it was not known whether these mAbs against L protein could detect all RABV strains. Therefore, we evaluated the reactivity of generated mAbs with different strains by comparing the conservation and variability of epitopes sequences recognized by mAbs. Amino acid alignments revealed epitope ^1724^VFNSL^1728^ recognized by mAb L-C is completely conserved among RABV strains, indicating that mAb L-C can detect all strains. Epitope ^1479^EIFSIP^1484^ is highly conserved among RABV strains of different strains except for a substitution (P1484S) in a single strain of China I sub-lineage in Asian lineage. The substitution P1484S eliminated the reactivity of ^1479^EIFSIP^1484^ with the mAb 3F3, indicating that mAb 3F3 can distinguish strains with P1484S substitution. However, the epitope ^1659^RALSK^1663^ was relatively less conserved with four substitutions (R1659Q, A1660T, K1663R, or K1663Q) in Asian (China I and China II), Africa-3, Cosmopolitan (China III) and Arctic-related (not including China IV) lineages. The A1660T substitution did not change the reactivity of mAb 3A6, while single substitutions (R1659Q, K1663R/Q) and dual substitutions, A1660T and K1663R, eliminated the reactivity of epitopes with mAb 3A6, suggesting mAb 3A6 could be used for differentiation of variable epitopes with R1659Q, K1663R, or K1663Q substitution in different lineages. The CVS-11 strain used in this study together with other vaccine strains clusters in the Cosmopolitan lineage (not China 3) in the phylogenetic tree, and the three epitopes of CVS-11 were completely conserved in other vaccine strains, suggested that the three mAbs could detect the vaccine strains.

Six conserved domains, CRI (230–422 aa), CRII (504–607 aa), CRIII (729–831 aa), CRIV (889–1057 aa), CRV (1090–1326 aa) and CRVI (1673–1747 aa), have been defined in L protein [23]. The conserved epitope ^1724^VFNSL^1728^ identified in this study was located in the conserved CRVI region, which possessed a K-D-K-E motif related to methyltransferase activities [24].while the location of the variable epitopes (^1479^EIFSIP^1484^ and ^1659^RALSK^1663^) in L protein falls in none of the conserved domains. The hydrophilic areas on the surface of the protein are more likely to arouse B-cell antigenicity [25].Structural analysis showed the three epitopes (^1479^EIFSIP^1484^ and ^1659^RALSK^1663^) were fully exposed on the surface of L protein, ^1724^VFNSL^1728^ were partially exposed on the surface and partially buried by the surrounding protein structure, which support the three epitopes had the antigenicity to produce antibody. In addition, the epitope ^1659^RALSK^1663^ recognized by 3F3 possessed high hydrophilicity index, antigenic index and surface probability, which also supports that this epitope has good antigenicity. However, these three indexes of epitopes ^1479^EIFSIP^1484^ and ^1724^VFNSL^1728^ are relatively low.

The L protein and P protein forming the viral RNA-dependent RNA polymerase (vRdRp), is crucial for virus replication [26]. The previous researches indicated the last 1562 to 2127 aa residues (especially the fragment ^1929^NPYNE^1933^) of L protein are essential for the P-L interaction [7,8]. Interestingly, the epitope ^1659^RALSK^1663^ targeted by mAb 3A6 and the epitope ^1724^VFNSL^1728^ targeted by mAb 3F3 both are a part of the last 566 aa located in the 1562 to 2127 aa of L protein. Thus, the epitope ^1659^RALSK^1663^ may be a potential target to block virus replication by disturbing the interaction of P and L proteins. In addition, the connector domain (CD) of L protein possesses eight helices, with no known enzymatic function [27], is perhaps critical to the structural organization. The epitope ^1479^EIFSIP^1484^ locates in the CD and is highly conserved, could perhaps function as a target blocking viral replication through altering the conformation of vRdRP. Reconstruction of polymerase experiments in vivo can be used to verify whether the mAbs can destruct the virus polymerase activity (such as disturbing the interaction of P and L protein) for inhibiting virus. On the other hand, the three mAbs seem to not enter into cells to inhibit virus; some strategies making mAbs into cells also should be considered.

The conservation of L is almost equal to N protein [28]. However, the traditional detection method of RABV is based on the detection of N protein, there are few detection or diagnostic method related to L protein [3]. We generated novel anti-RABV L protein monoclonal antibodies (3F3, 3A6 and L-C) and identified three epitopes, including a novel conserved antigenic epitope ^1724^VFNSL^1728^ recognized by mAb L-C and two novel variable epitopes (^1479^EIFSIP^1484^ and ^1659^RALSK^1663^) recognized by mAbs 3F3 and 3A6, respectively, which revealed the different reactivity ability of the three mAb with RABV strains. Thus, three mAbs could be used to detect or differentiate RABV strains. These results may facilitate future studies in the structure and function of RABV L protein, as well as development of new detection methods for RABV infection.

## Figures and Tables

**Table 1:** Oligonucleotide primers used for ampliying the truncated L gene by polymerase chain reaction (PCR).

Amino Acid Fragment	Sequence (5′–3′)
aa 1431-1558	gctgatatcggatccgaattcTCGATTTGCTTCTTGACACG (*Eco*RI)
	ctcgagtgcggccgcaagctTTTACAAGTTTCTCTCAACCCTCTG (*Hin*dIII)
aa 1520–1665	gctgatatcggatccgaattcTCCCCGGAGAATGACTGGCTGT (*Eco*RI)
	ctcgagtgcggccgcaagcttTTAAAACCTCTTAGAGAGGGCCCTAATG (*Hin*dIII)
aa 1627–1754	gctgatatcggatccgaattcTCCCACAAGGCAGGATGTTCAG (*Eco*RI)
	ctcgagtgcggccgcaagcttTTAGATGTCATCTCCTCCACTCATG (*Hin*dIII)
aa 1627–1675	ccgcgtggatccccggaattcTCCCACAAGGCAGGATGTTCAG (*Eco*RI)
	gtcacgatgcggccgctcgagCACTCTCAGGCCCGAGATCAAG (*Xho*I)
aa 1666–1720	ccgcgtggatccccggaattcCAAAACCCCTTGATCTCGG (*Eco*RI)
	gtcacgatgcggccgctcgagATCTGGAAACATGTCGAGAAC (*Xho*I)
aa 1711–1754	ccgcgtggatccccggaattcTCAAGGGCAGTTCTCGACATG (*Eco*RI)
	gtcacgatgcggccgctcgagGATGTCATCTCCTCCACTCATG (*Xho*I)

**Table 2:** Synthetic complementary oligonucleotide sequences for expressing short peptides less than 20 amino acids.

Amino Acid Fragment	Sequence (5′–3′)
aa 1477–1492	aattcAGAGGAGAAATATTCTCTATCCCTCAGAAAATCCCCGCCGCTTACCCAc
	tcgagTGGGTAAGCGGCGGGGATTTTCTGAGGGATAGAGAATATTTCTCCTCTg
aa 1486–1501	aattcAAAATCCCCGCCGCTTACCCAACCACTATGAGAGAAGGCAACAGATCGc
	tcgagCGATCTGTTGCCTTCTCTCATAGTGGTTGGGTAAGCGGCGGGGATTTTg
aa 1514–1529	aattcCGAGAGGCAATCACGGCGTCCCCGGAGAATGACTGGCTGTGGATCTTCc
	tcgagGAAGATCCACAGCCAGTCATTCTCCGGGGACGCCGTGATTGCCTCTCGg
aa 1478–1484	aattcGGAGAAATATTCTCTATCCCTc
	tcgagAGGGATAGAGAATATTTCTCCg
aa 1479–1483	aattcGAAATATTCTCTATCc
	tcgagGATAGAGAATATTTCg
aa 1479–1485	aattcGAAATATTCTCTATCCCTCAGc
	tcgagCTGAGGGATAGAGAATATTTCg
aa 1480–1484	aattcATATTCTCTATCCCTc
	tcgagAGGGATAGAGAATATg
aa 1479–1484	aattcGAAATATTCTCTATCCCTc
	tcgagAGGGATAGAGAATATTTCg
aa 1627–1642	aattcTCCCACAAGGCAGGATGTTCAGAATGGGTCTGCTCTGCTCAACAGATTc
	tcgagAATCTGTTGAGCAGAGCAGACCCATTCTGAACATCCTGCCTTGTGGGAg
aa 1638–1653	aattcTCTGCTCAACAGATTGCCGTCTCCACCTCAGCCAACCCGGCTCCTGTCc
	tcgagGACAGGAGCCGGGTTGGCTGAGGTGGAGACGGCAATCTGTTGAGCAGAg
aa 1650–1665	aattcCCGGCTCCTGTCTCAGAGCTTGACATTAGGGCCCTCTCTAAGAGGTTTc
	tcgagAAACCTCTTAGAGAGGGCCCTAATGTCAAGCTCTGAGACAGGAGCCGGG
aa 1657–1665	aattcGACATTAGGGCCCTCTCTAAGAGGTTTc
	tcgagAAACCTCTTAGAGAGGGCCCTAATGTCg
aa 1658–1665	aattcATTAGGGCCCTCTCTAAGAGGTTTc
	tcgagAAACCTCTTAGAGAGGGCCCTAATg
aa 1659–1663	aattcAGGGCCCTCTCTAAGc
	tcgagCTTAGAGAGGGCCCTg
aa 1660–1663	aattcGCCCTCTCTAAGc
	tcgagCTTAGAGAGGGCg
aa 1659–1662	aattcAGGGCCCTCTCTc
	tcgagAGAGAGGGCCCTg
aa 1723–1736	aattcCTTGTGTTCAACAGCCTATTGGAGGTGAATGATCTGATGGCTc
	tcgagAGCCATCAGATCATTCACCTCCAATAGGCTGTTGAACACAAGg
aa 1729–1744	aattcTTGGAGGTGAATGATCTGATGGCTTCCGGAACACATCCACTGCCTCCTc
	tcgagAGGAGGCAGTGGATGTGTTCCGGAAGCCATCAGATCATTCACCTCCAAg
aa 1737–1750	aattcTCCGGAACACATCCACTGCCTCCTTCAGCAATCATGAGTGGAc
	tcgagTCCACTCATGATTGCTGAAGGAGGCAGTGGATGTGTTCCGGAg
aa 1723–1728	aattcCTTGTGTTCAACAGCCTAc
	tcgagTAGGCTGTTGAACACAAGg
aa 1724–1728	aattcGTGTTCAACAGCCTAc
	tcgagTAGGCTGTTGAACACg
aa 1725–1730	aattcTTCAACAGCCTATTGGAGc
	tcgagCTCCAATAGGCTGTTGAAg
aa 1723–1727	aattcCTTGTGTTCAACAGCc
	tcgagGCTGTTGAACACAAGg
P1484S	aattcGAAATATTCTCTATCTCTc
	tcgagAGAGATAGAGAATATTTCg
K1663R	aattcAGGGCCCTCTCCAGGc
	tcgagCCTGGAGAGGGCCCTg
A1660T	aattcAGGACCCTCTCTAAGc
	tcgagCTTAGAGAGGGTCCTg
A1660T + K1663R	aattcAGGACCCTCTCTAGAc
	tcgagTCTAGAGAGGGTCCTg
K1663Q	aattcAGGGCTCTCTCCCAGc
	tcgagCTGGGAGAGAGCCCTg
R1659Q	aattcCAGGCCCTATCTAAGc
	tcgagCTTAGATAGGGCCTGg

Restriction enzyme sites introduced in primer pairs are in lowercase font.

## Data Availability

Data sharing not applicable.
